# The comprehensive impact of thermal-PM2.5 interaction on subjective evaluation of urban outdoor space: A pilot study in a cold region of China

**DOI:** 10.1371/journal.pone.0304617

**Published:** 2024-05-31

**Authors:** Dahu Lin, Sujing Gao, Meng Zhen

**Affiliations:** 1 School of Architecture and Art, Hebei University of Architecture, Zhangjiakou, 075000, Hebei, China; 2 School of Sciences for the Human Habitat, University of the Chinese Academy of Sciences, Beijing, 100000, China; 3 School of Human Settlements and Civil Engineering, Xi’ an Jiaotong University, Xi’ an, Shaanxi, 710049, China; Kanazawa University, JAPAN

## Abstract

Urban outdoor space has a very important impact on the quality of people’s outdoor activities, which has influenced people’s health and moods. Its influence is the result of the combined action of various factors. Thermal and air quality environment are important factors affecting the overall comfort of the urban outdoor space. At present, there are few research on interaction with thermal and air quality environment. Therefore, a meteorological measurement and questionnaire survey have been conducted in a representative open space in a campus in Xi’an, China. The following are the research results:(1) Mean physiological equivalent temperature (MPET) is a significant factor affecting thermal sensation vote (TSV) and thermal comfort vote (TCV). PM2.5 has no significant effect on thermal comfort vote (TCV), but it is a considerable factor affecting thermal sensation vote (TSV) when 10.2°C ≤ MPET<21°C (P = 0.023 *). (2) PM2.5 is a significant factor affecting air quality vote (AQV) and breathing comfort vote (BCV).Mean physiological equivalent temperature (MPET) has no significant impact on air quality vote (AQV), but it is a considerable factor affecting breathing comfort vote (BCV) when 10.2°C ≤ MPET<21°C (P = 0.01 **). (3) Mean physiological equivalent temperature (MPET) is a significant factor affecting overall comfort vote (OCV), but PM2.5 is not. In general, When 10.2°C ≤ MPET<21°C (-0.5 < -0.37 ≤ TCV ≤ 0.12 <0.5), the interaction between thermal and PM2.5 environment is significant on thermal sensation vote (TSV) and breathing comfort vote (BCV). This study can provide experimental support for the field of multi-factor interaction, which has shown that improving the thermal environment can better breathing comfort, while reducing PM2.5 concentration can promote thermal comfort. And can also provide reference for the study of human subjective comfort in urban outdoor space in the same latitude of the world.

## 1 Introduction

With the continuous development of economy and society, people pay more and more attention to subjective comfort. It is found that subjective comfort is one of the most critical factors in the application of public space. Exploring the influencing factors of subjective comfort and finding ways to improve subjective comfort has become a growing concern of the academic community [1]. Urban outdoor space is an important asset and plays a significant role in people’s daily life. Many behavior-based researches are based on it [2]. Urban outdoor space will affect all aspects of people’s daily life, such as health, social contact, and even emotions [3]. Different from the relatively stable indoor microclimate environment, the subjective comfort of the human body in the urban outdoor space will be affected by a variety of factors and show complexity [4]. Therefore, it is necessary to research the impact of various factors on human environmental assessment. In recent years, many scholars at home and abroad have carried out a lot of research from multiple perspectives that may affect the subjective comfort of human body in urban outdoor space, including thermal environment, air quality environment, light environment, acoustic environment, wind environment, physiological and psychological adaptation, regional differences and gender differences [3–6].

### 1.1 Study of subjective thermal comfort under interaction

In the current study of human subjective comfort, numbers of studies focus on human subjective thermal comfort, and a considerable number of studies on the field of the interaction between thermal and other environments. Nitidara et al. found that the visual sensation under the sun will rise with the increase of thermal sensation. The auditory sense will go up with the increase of thermal sense and noise level. And the change of auditory factor in visual factor, auditory factor and thermal factor has the greatest impact on the comfort of public open space [1]. Zhang et al. proposed that emotions have an impact on people’s subjective thermal sensation [3]. Wang and Liu found that emotional state has no significant impact on physiological parameters during exercise, but shows the completely opposite result during standing and sitting posture [7]. Ba and Kang discovered that in terms of thermal evaluation, higher temperature will lead to a significant increase in thermal sensation in all seasons, while traffic noise will only lead to a slight increase in thermal sensation in summer. At the same time, the thermal comfort of the whole year is affected by temperature and traffic noise. The higher the traffic noise, the lower the thermal comfort [5]. Jin et al. found out that in summer, the thermal evaluation of subjects can be effectively improved by birdsong and slow dance music, while the sound of dogs barking, talking and traffic noise with high sound level will lead to the decline of thermal evaluation. In the transition season, all types of sound will lead to a decrease in thermal evaluation [8]. Lau et al. identified that human thermal tolerance is higher in quiet and beautiful outdoor environment, while human thermal sensitivity is lower in quiet and beautiful outdoor environment [9].

### 1.2 Research on breathing comfort

Nowadays, there are few researches on the breathing comfort of urban outdoor space, and most of them use the actual measurement method to directly study the air quality rather than subjective comfort. Wu et al. adopted the methods of field measurement and wind tunnel experiment, and found that wind speed (54.2%), vehicle flow (27.7%), temperature (14.2%) and time factor (7.6%) all affect PM2.5 concentration, of which wind speed has the greatest impact on PM2.5 concentration [10]. Wang et al. found that there is a dynamic and complex relationship between air quality and urban form. Continuous and compact urban form, reasonable building layout, reasonable population density, reasonable road density and high green space rate are all beneficial methods for improving urban air quality [11]. Xie et al. obtained concentration observations from 18 air quality monitoring stations in Shenzhen and concluded that the accumulation patterns of PM2.5 and NO_2_ are different. High concentration of PM2.5 is often related to external transportation, while high concentration of NO_2_ is caused by local accumulation [12]. Zhang et al. found that the air quality in China is affected by the region rather than the city level; The CO_2_ emissions are affected by the city category rather than the region, and the proposal of more tough emission reduction targets can reduce the concentration of PM2.5 [13]. Fu et al. found out that the concentration of particulate matter in the building will be significantly affected by human activities such as window-opening behavior, and the smaller particles are easier to penetrate the building facade than the larger particles [14]

Moreover, there are few research on the interaction between thermal and air quality environment, and only a few studies start from the comprehensive impact of the two. Miao et al. studied the vertical synergy between air quality and thermal comfort in urban street space. The study not only showed that O_3_ increased exponentially with the increase of PET, but also showed that with the increase of PET, NO_2_ increases linearly at the bottom of the street but decreases at the top of the street [15]. The current bioclimate and air quality index do not provide enough information to explain the comprehensive impact of outdoor space on human physiology, Fahad et al. combined thermal comfort and air quality into outdoor comfort index, and the universal thermal climate index (UTCI) which is derived at hourly intervals and the air quality index (AQI) obtained by the Environmental Protection Agency (EPA) observation station are combined into a framework to conduct relevant research on the comprehensive impact of thermal comfort and air quality [16]. Steeneveld et al. combined air quality and thermal comfort to judge urban climate [17]. Jacobs et al. determined the outdoor "biological comfort" threshold by combining thermal stress, air particle concentration and grass pollen count, although other researchers have studied the individual effects of different variables, this is the first time to propose a unified biological comfort threshold [18].

### 1.3 Innovation and contribution of this study

At present, there are a certain quantity of research on the quality of urban outdoor space and a few research on human subjective breathing comfort [19–22]. In addition, the urban haze pollution in the institute is prominent, and the thermal and PM2.5 environment are all important factors that affect the human subjective comfort of urban outdoor space [23,24]. The state of the two factors under the comprehensive environment is attracted to people’s widespread attention and has an important impact on health. Accordingly, it is very meaningful to combine thermal environment and air quality. The purpose of this research: (1) Study the interaction between thermal environment and PM2.5 (2) Study the thermal evaluation, air quality evaluation and overall comfort evaluation under the interaction of thermal environment and PM2.5. Studying the interaction between multiple factors can help urban designers better plan and solve real-world problems. By controlling one factor to help improve the subjective comfort of another. Some places often improve thermal comfort in summer by installing sprinkler facilities, shading facilities, etc. These measures can not only improve thermal comfort but also improve breathing comfort. Besides, when dealing with patients with sudden symptoms such as shortness of breath outdoors, in addition to necessary scientific treatment, we can also help alleviate their symptoms by creating a more comfortable thermal environment. This study provides a reference for improving the environmental comfort of urban outdoor space, provides experimental support for building a healthy city, and provides a reference for the study of human subjective comfort of urban outdoor space in the same latitude of the world, which has theoretical and practical significance.

## 2. Methods

### 2.1 Location

**Fig 1:**
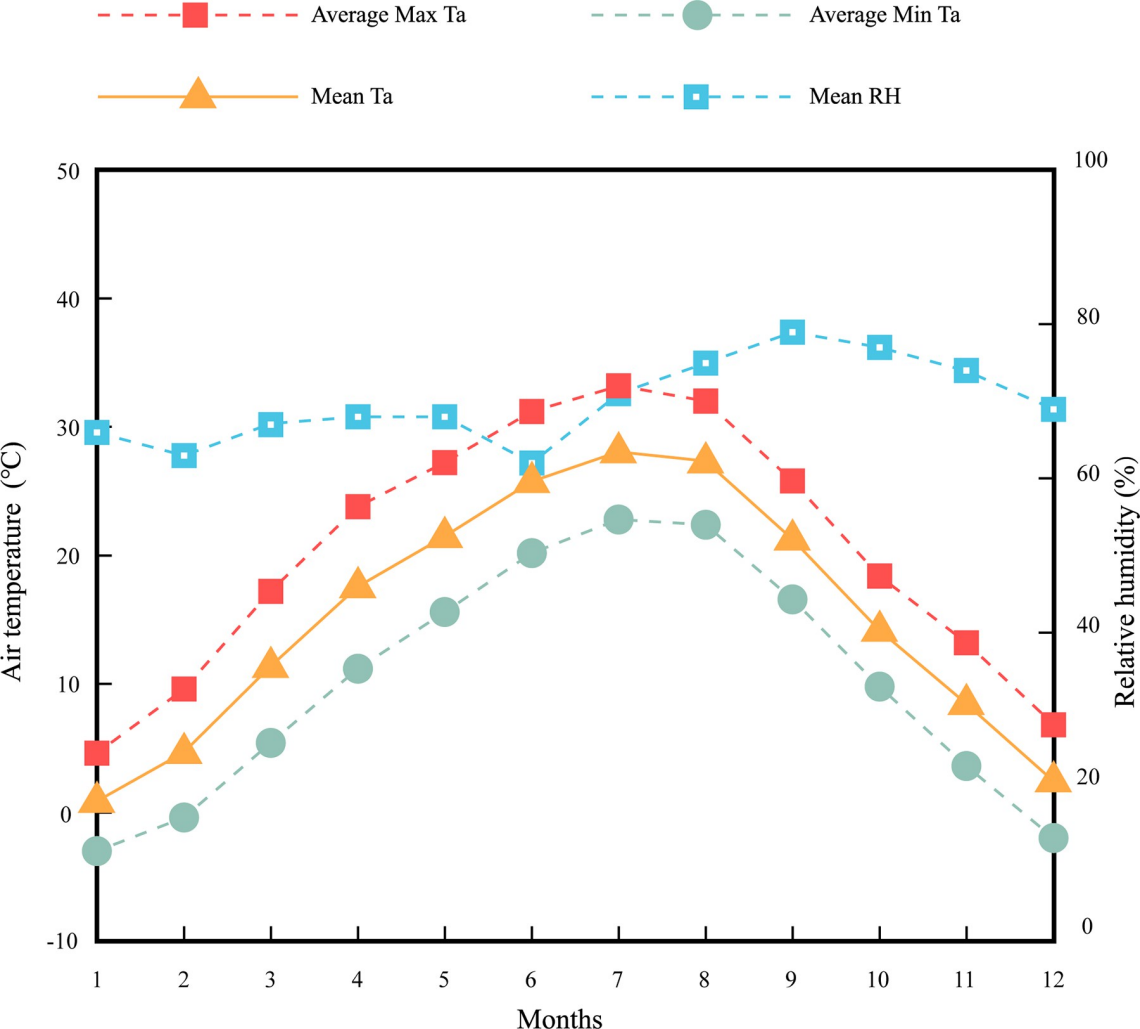
Monthly mean/average maximum/ average minimum Ta and mean RH from 2016 to 2020 in Xi’an, China.

Xi’an has a warm temperate semi-humid continental monsoon climate, belonging to the cold zone. The temperature range is relatively large every year, with abundant sunshine. Spring and autumn are short, and the temperature changes are intense. In spring, there is little rain and snow, and there are often strong winds, sandstorms, and hail and thunderstorms in summer and autumn. Fig 1 shows the basic temperature and humidity situation of Xi’an City from 2016 to 2020, the average annual temperature of Xi’an is 15.2° C. The hottest and coldest months in Xi’an are July and January, with an average temperature of 28° C and 0.8° C, respectively. From April to May, there were often sand and dust in Xi’an. The average temperature in April was 17.5° C, with an average relative humidity of 68%. The average temperature in May was 21.4° C, and the average relative humidity was 68%.

**Fig 2:**
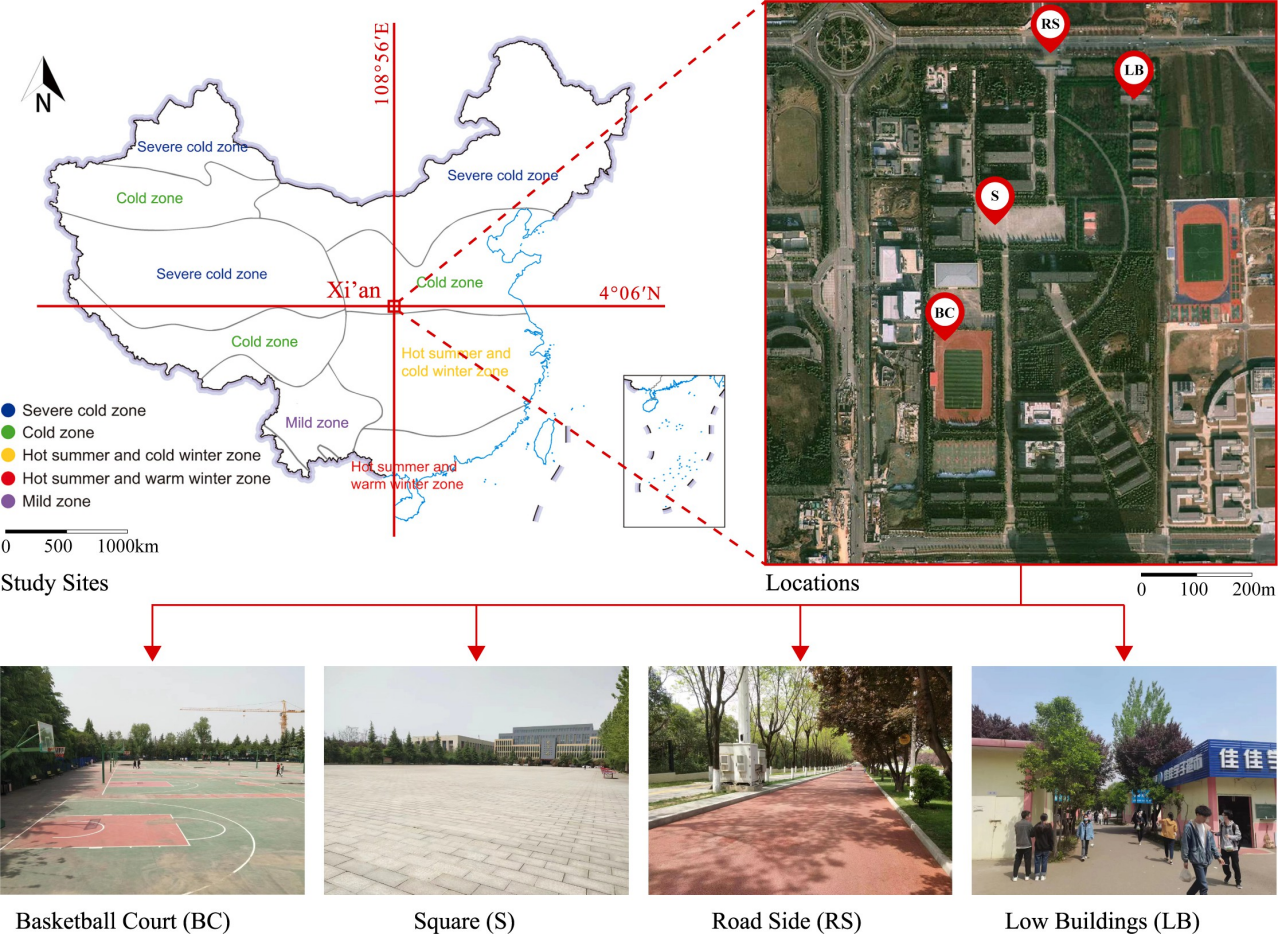
Locations.

There are various outdoor venues such as open squares, forests, and architectural complexes on Xi’an Vocational University (4°06′N, 108°56′E). This study selected four typical locations, including road side (RS), low buildings (LB), square (S) and basketball court (BC), as clearly seen from Fig 2.

### 2.2 Questionnaire

The questionnaire of this study includes three parts: the respondents’ personal information, the respondents’ subjective feelings and the research environment, and all abbreviations used in this study are shown in Table 1. The respondents’ personal information includes age, gender, nationality, education level, occupation, health, etc. And subjective feelings of respondents include TSV, TCV, OCV, AQV and BCV in the current environment. Among them, the overall comfort OCV refers to the overall subjective feeling of the human body in the thermal-PM2.5 environment; The research environment refers to the physical information recorded by the investigators according to the actual situation, such as the survey location, the survey time, the movement status of the survey object, the clothing and the current environment temperature, humidity, wind speed, black ball temperature, PM2.5, etc.

**Table 1:** Abbreviations.

Full name	Abbreviation
Thermal Sensation Vote	TSV
Thermal Comfort Vote	TCV
Overall Comfort Vote	OCV
Air Quality Vote	AQV
Breathing Comfort Vote	BCV
Physiological Equivalent Temperature	PET
Mean Physiological Equivalent Temperature	MPET
Wind speed	V_a_
Relative humidity	RH
Black bulb temperature	T_mrt_
Global radiation	G

To make the research conclusion more detailed and reliable, this study uses a 7-point scale to measure people’s subjective evaluation of the thermal environment, PM2.5 and the overall environment [4]. Table 2 shows the specific 7-point scale.

**Table 2:** 7-point scale.

Score	TSV	TCV	OCV	AQV	BCV
-3	Cold	Very uncomfortable	Very uncomfortable	Very bad	Very uncomfortable
-2	Cool	Uncomfortable	Uncomfortable	Bad	Uncomfortable
-1	Slightly cool	Slightly uncomfortable	Slightly uncomfortable	Slightly bad	Slightly uncomfortable
0	Neutral	Neutral	Neutral	Neutral	Neutral
1	Slightly warm	Slightly comfortable	Slightly comfortable	Slightly good	Slightly comfortable
2	Warm	Comfortable	Comfortable	Good	Comfortable
3	Hot	Very comfortable	Very comfortable	Very good	Very comfortable

### 2.3 Measurement

The study was conducted at four typical sites on the campus of Xi’an Vocational University of Information. We require participants to stand still for 10 minutes to reduce the impact of activities on subjective thermal comfort and adapt to the environment at the same time. The research subject of this study is adults. The study obtained verbal consent from all participants. Our researchers will explain the nature, purpose, potential benefits, and risks of the study to the participants before filling out the questionnaire and the questionnaire will also provide corresponding explanations. All participants filled out the questionnaire with informed consent, which serves as proof of our verbal consent. A weather station at each site was installed 1.2 m above the ground to record meteorological data. These measures were temperature (T_a_), black sphere temperature (T_mrt_), relative humidity (RH), wind speed (V_a_), global radiation (G) and PM2.5. Table 3 shows the basic parameters of measuring instruments that used in this study. The instrument was selected according to ISO 7730 [25], which was consistent with the accuracy and sensitivity of the test. All data in the paper comes from survey questionnaires. We will remove invalid questionnaires and conduct subsequent analysis and calculation on the data from valid questionnaires. Table 4 reports the variable data including temperature (T_a_), relative humidity (RH), wind speed (V_a_), and black sphere temperature (T_mrt_). And all of them would be put into Rayman model to calculate PET [4], then used for subsequent research after averaging on a daily basis

**Table 3:** Basic parameters of measuring instruments.

Instrument	Meteorological parameter	Measuring range	Measurement accuracy
TSI 8530/8530EP	PM2.5	0.001–400 mg/m^3^; 0.001–150 mg/m^3^;	±0.1%
JT2022WBGT Index Monitor	Relative humidity (RH) Black bulb temperature (T_b_)	15–95%RH -20°C-125°C	±3% ±0.5°C
JT1402 Wind speed sensor	Wind speed (V_a_)	0.05-20m/s	±0.05 m/s(0.05~5.0m/s) ±0.1 m/s(5.0~20.0m/s)
JTR05 Solar radiation tester	Global radiation (G)	0-2000W/ m^2^	±2%

**Table 4:** Variable data.

Year	Month	Day	Ta (°C)	RH(%)	Va(m/s)	Tmrt (°C)	PM2.5
2021	April	5	13	45.8	0.7	12.9	65
		6	15.2	45.8	0.7	12.9	65
		7	14.8	54.2	0.4	15.3	92.9
		8	20.1	34.7	0.3	24.2	109
		14	14	61.9	0.7	16.4	84.5
		17	13	37.6	0.5	15.2	88.9
		21	19	34.7	0.2	16	90
		22	19.4	61.5	0	17.8	69.4
		23	15.7	86.4	0	15.9	114.6
		24	16	87.0	0	16.1	117.2
	May	1	26.5	18.0	0.6	26.1	62.9
		4	25.3	17.2	0.7	25.3	68.2
		6	28	13.6	0.2	24.2	40
		7	28	13.6	0.2	24.2	40
		11	18	51.1	0.5	26.1	90
		13	20	61.9	0.1	20.8	140.4
		20	34	50	1	35.7	78.6
		22	20.5	43.9	1.7	23	98
		23	21	43.9	1.3	24.8	80
		24	21	43.9	1.3	24.8	80
		28	21	56	0.5	24.9	70

### 2.4 Subject

This study was conducted continuously from April 5, 2021, to May 29, 2021, and a total of 774 valid questionnaires were collected, including 436 in April and 338 in May. The basic information of the interviewees is shown in Table 5. As for the occupational distribution, the study site is on campus, and the main subjects are students and teachers. There are only a few self-employed people and medical workers such as doctors among the interviewees, among which students account for 91.86% and teachers account for 5.94%. Regarding age distribution, the age distribution of the sample is wide, and the age group of 18 to 20 years old is mainly concentrated, accounting for 68.73%. In terms of gender distribution, male respondents accounted for 68.22%. As for the education level of respondents, 94.83% of them have received higher education.

**Table 5:** Basic information of interviewees.

Age	18–20	21–25		26–30		31–35
	68.73%	27.78%		2.33%		1.16%
Gender	male			female		
	68.22%			31.78%		
Educational level	Senior high school and below		Junior college		Undergraduate	
	5.17%		60.98%		33.85%	
Occupation	student	teacher		Medical personnel		Self-employed and self-employed
	91.86%	5.94%		1.94%		0.26%
Health condition	healthy		ill		general	
	72.48%		3.75%		23.77%	

### 2.5 Procedures

In terms of air quality environmental evaluation index, PM2.5 is used as the evaluation index, and the actual measurement range of PM2.5 in this study is40μg/*m*^3^≤PM2.5≤140.4μg/*m*^3^. Combining the distribution of data and the air quality grades guidelines of China, as clearly showed in Table 6 [26,27]. Table 7 illustrates the specific grouping. The study divided the PM2.5 values into the following three groups: PM2.5≤70μg/*m*^3^, 70<PM2.5≤90μg/*m*^3^ and PM2.5>90μg/*m*^3^.

**Table 6:** Standard Values of 24-hour PM2.5 Average of Air Quality Grade in China.

Grade	excellent	good	Mild pollution	Moderate pollution	Severe pollution	Serious pollution
PM2.5	0~35μg/m^3^	35~75μg/m^3^	75~115μg/m^3^	115~150μg/m^3^	150~250μg/m^3^	>250μg/m^3^

**Table 7:** Grouping situation.

Index	grouping			
PM2.5	PM2.5≤70μg/*m*^3^	70 <PM2.5≤ 90μg/*m*^3^		PM2.5>90μg/*m*^3^
MPET	10.2°C≤MPET<21°C		21°C≤MPET≤35°C	

**Fig 3:**
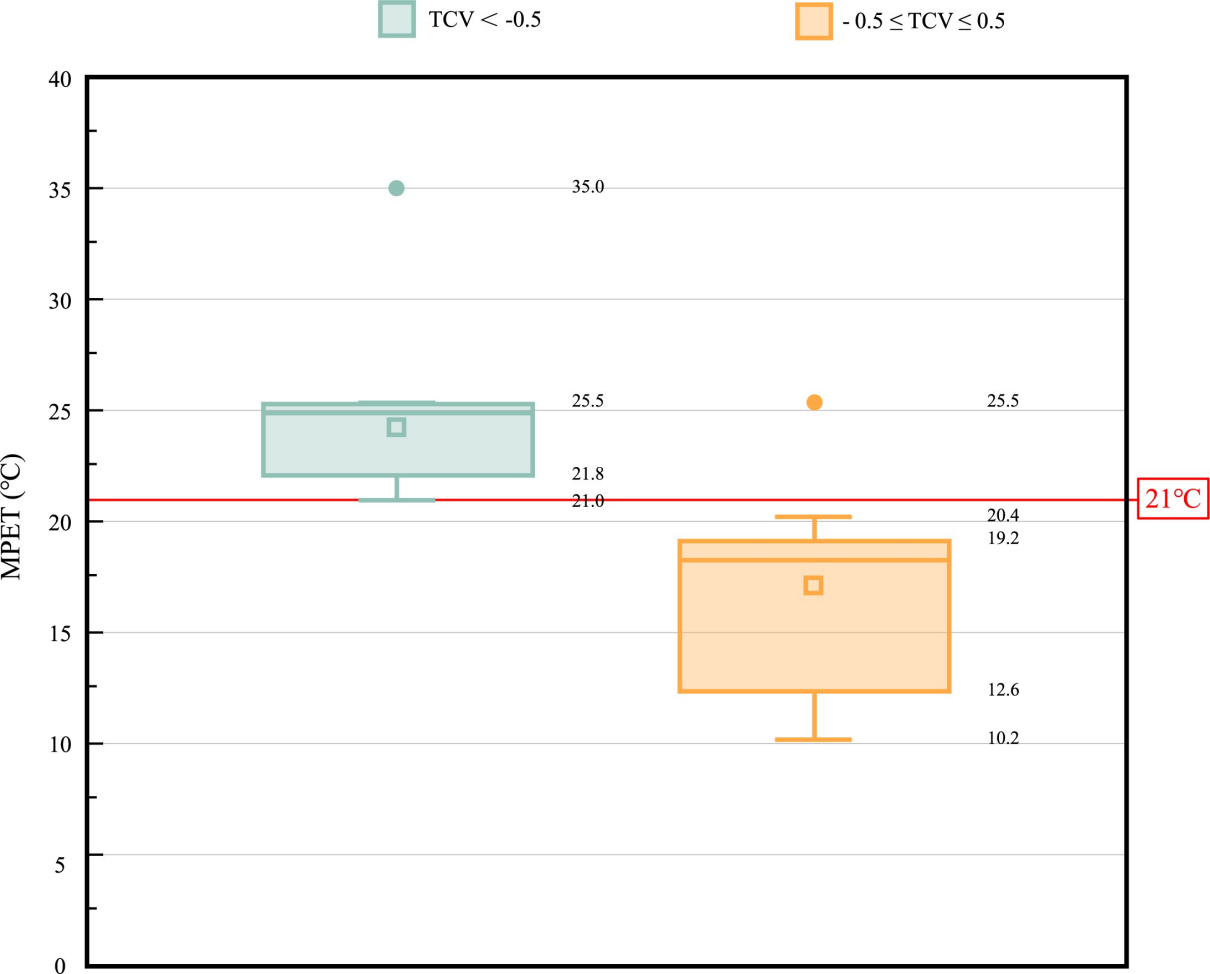


In terms of evaluation indicators of thermal environment, the interviewees in this study are located outdoors, which are affected by a variety of complex micro-climates. PET is defined as the air temperature at which the body’s heat budget in a typical indoor environment (without wind and solar radiation) is balanced by the same core and skin temperature as the complex outdoor conditions which is to be assessed. In this way, PET enables the public to compare the overall impact of complex thermal conditions outside with his or her own experience indoors [28]. PET is a heat index derived from the energy balance of the human body, which is very suitable for evaluating the thermal composition of different climates [29]. At present, the evaluation standard of thermal comfort is mainly PET model, which integrates the air temperature and humidity, wind speed, solar radiation, people’s wearing and sports conditions and so on, so it is relatively accurate and widely used. Therefore, PET was selected in this study to evaluate the physiological effects of thermal environment, and the MPET in the study was the result of daily average PET. The calculation results of Rayman shows that the MPET ranges from 10.2C 8 to 35C. Fig 3 shows the MPET corresponding to different TCV ranges, which is also the reason of grouping. According to preliminary data statistical analysis, when TCV < -0.5, the human body feels a bit uncomfortable with heat, and at this time, 21°C ≤ MPET ≤ 35°C; When -0.5 ≤ TCV ≤ 0.5, the human body feels comfortable with heat, and at this time, except for one data, 10.2°C ≤ MPET < 21°C. Therefore, the study chose MPET = 21°C as the boundary point and divided MPET into two groups: 10.2°C ≤ MPET < 21°C and 21°C ≤ MPET ≤ 35°C, as clearly illustrated in Table 7.

## 3. Results

### 3.1 Comprehensive impact on thermal evaluation

Linear regression analysis was used to analyze whether MPET and PM2.5 had significant effects on TSV and TCV. After correlation analysis of all data with SPSS, the results are shown in Table 8. The research results show that MPET has a very significant impact on both TSV and TCV, with P values of 0.000 * *, which are significant at the level of 0.001, while PM2.5 has no very significant impact on both TSV and TCV, with P values of 0.857 and 0.239, respectively. The data within the range of 10.2°C≤ MPET <21°C were analyzed by SPSS. The results are shown in Table 9. The research results show that MPET has a very significant impact on TSV and TCV, with P values of 0.000 * * and 0.001 * *, respectively, which are significant at the level of 0.001, while PM2.5 only has a significant impact on TSV, with P value of 0.023 *, which is significant at the level of 0.05, but PM2.5 has no significant impact on TCV, with P value of 0.067.

**Table 8:** Importance and fitting equation of indicators under TSV and TCV.

		MPET	PM2.5
TSV	P	0.000***	0.857
	R^2^	0.753	0.002
	F	57.964	0.033
	equation	y = 0.0599x - 1.1713	y = -0.0007x +0.0396
TCV	P	0.000***	0.239
	R^2^	0.765	0.072
	F	61.791	1.48
	equation	y = -0.0504x + 0.6828	y = 0.0037x - 0.5937

**Table 9:** Importance and fitting equation of indexes under TSV and TCV when 10.2°C ≤ MPET<21°C.

		MPET	PM2.5
TSV	P	0.000***	0.023*
	R^2^	0.663	0.338
	F	25.593	6.629
	equation	y = 0.0842x - 1.5594	y = 0.0101x - 1.0974
TCV	P	0.001***	0.067
	R^2^	0.584	0.235
	F	18.247	4.002
	equation	y = -0.04x + 0.5411	y = -0.0043x + 0.273

**Fig 4:**
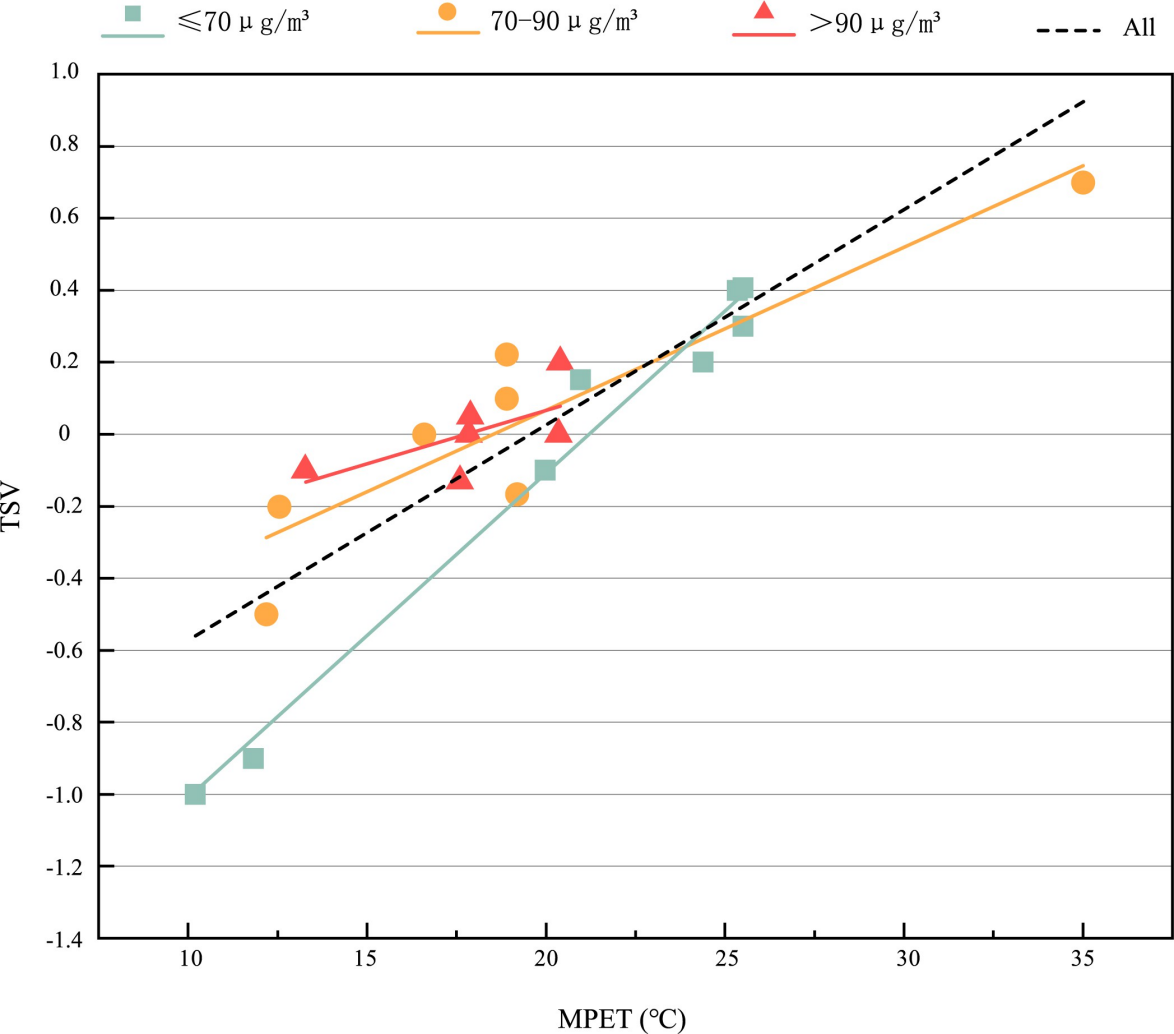
TSV value under different thermal-PM2.5 environments.

**Fig 5:**
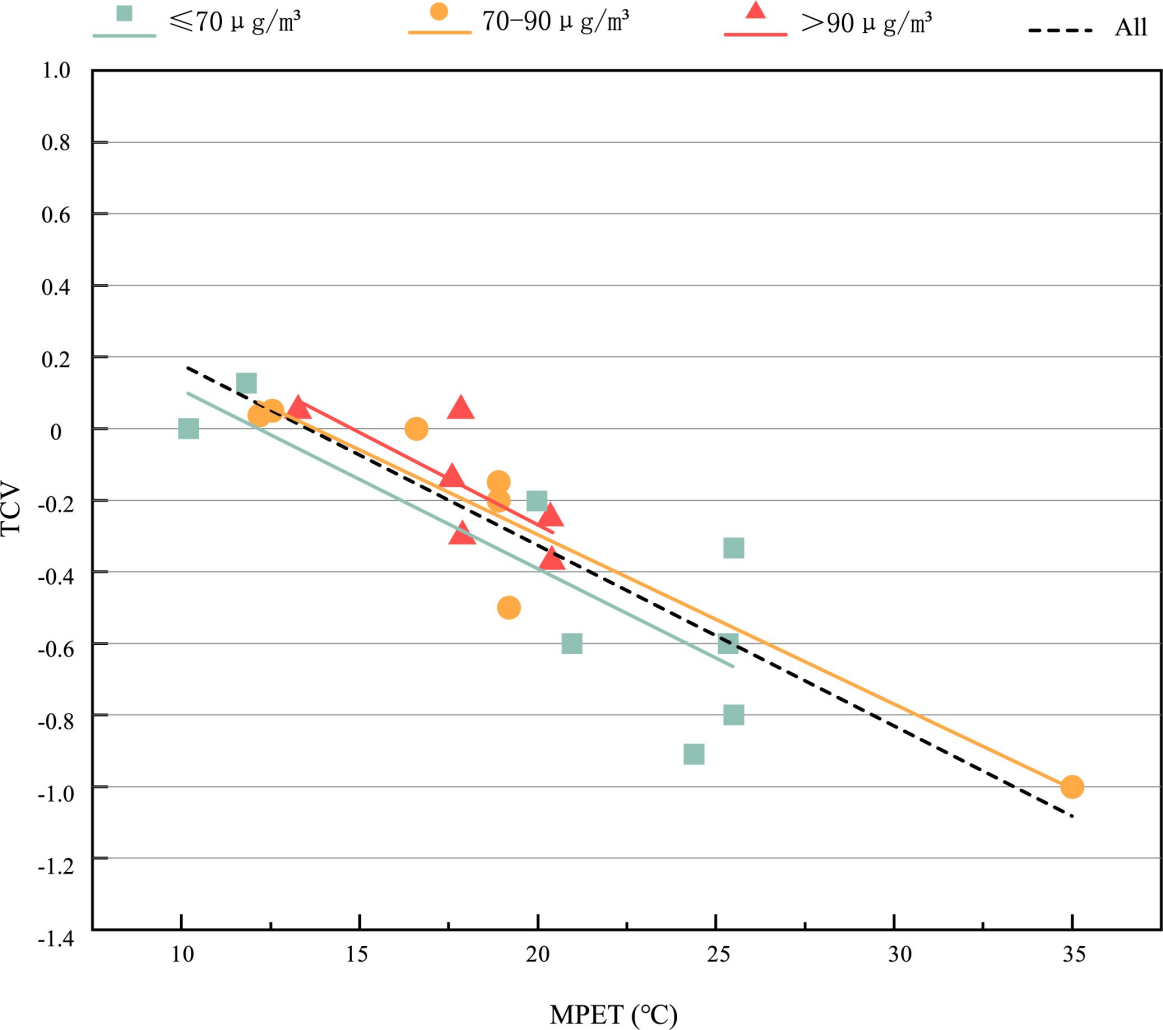
TCV values under different thermal-PM2.5 environments.

Fig 4 shows the subjective TSV of the respondents in the thermal-PM2.5 interaction. The range of MPET is 10.2°C~35°C, the TSV value is—1~0.7, and the average value is—0.02. When MPET is lower than 19.55°C, the TSV evaluation is often cold. In general, the TSV gradually increases with the increase of MPET. At the same time, we found that in the range of 10.2°C ≤ MPET<21°C, that is, when people feel slightly cold, different PM2.5 groups have obvious differences. Under the same MPET, the higher PM2.5, the higher TSV, the average value of TSV under the environment of ≤70μg/*m*^3^, 70~90μg/*m*^3^ and >90μg/*m*^3^ is -0.46, -0.09 and 0.003 respectively, with a trend of rising. It shows that when 10.2°C ≤ MPET<21°C, the higher the PM2.5, the stronger the respondents’ thermal sensation. The increase of PM2.5 will increase the respondents’ thermal sensation. There is interaction between thermal environment and PM2.5. Fig 5 shows the respondents’ subjective TCV under different thermal-PM2.5 environments. The range of MPET is 10.2°C~35°C, the TCV value is—1~0.13, and the average value is—0.29, indicating that the respondents feel a little uncomfortable in terms of thermal comfort. In general, TCV gradually decreases with the increase of MPET. The possible reason may be that in April and May of this study, the temperature in Xi’an gradually increases, the weather is gradually hot, and people’s thermal comfort gradually decreases with the increase of MPET. In this figure, different PM2.5 groups are slightly different but not obvious, the average value of TCV under the environment of ≤70μg/*m*^3^, 70~90μg/*m*^3^ and >90μg/*m*^3^ is -0.025, -0.127 and 0.160 respectively, with a slight trend of rising. It shows that PM2.5 has an impact on TCV, but it is not significant. TCV is mainly affected by MPET. The first reason may be that the concentration range of PM2.5 is 60μg/*m*^3^~140.37μg/*m*^3^, the maximum value of PM2.5 concentration is 140.37μg/*m*^3^, it belongs to light pollution. In addition, all PM2.5 concentrations are excellent or good, and the air quality is relatively good. Respondents are not sensitive to the change of PM2.5 concentration in the range of 60μg/*m*^3^~140.37μg/*m*^3^. Moreover, the interviewees are not sensitive to the change of PM2.5 because they have been living in haze weather for a long time and have had adaptability.

**Fig 6:**
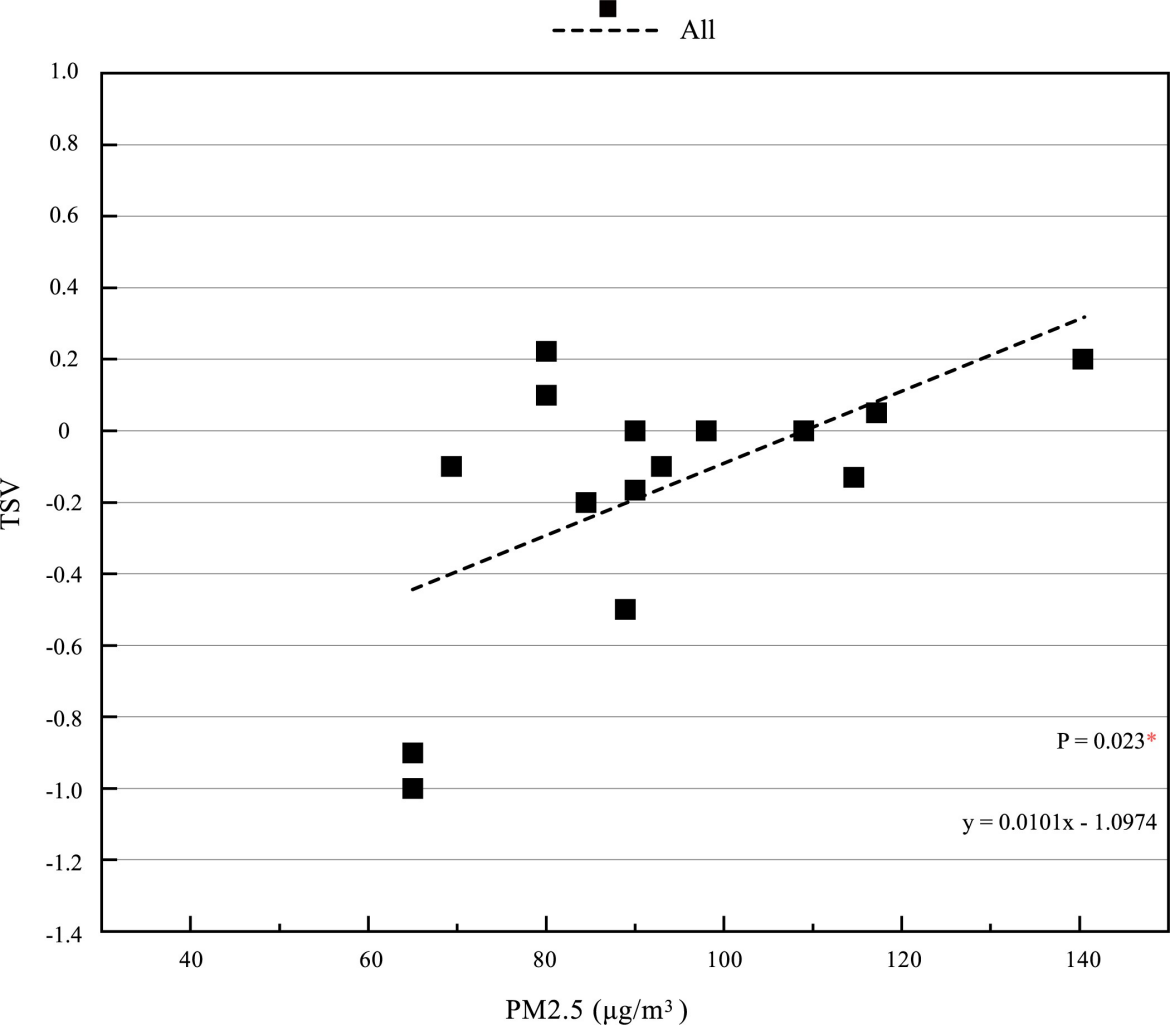
TSV value under different thermal-PM2.5 environments when 10.2°C ≤ MPET < 21°C.

During the study, we found the critical point of 21°C. In order to further study and prove the impact of the interaction between thermal and PM2.5 environment on the subjective comfort of human body, the data in the range of 10.2°C ≤ MPET<21°C were continued to be analyzed by SPSS. The results showed that when 10.2°C ≤ MPET<21°C, the correlation between TSV and PM2.5 also showed significant, with a P value of 0.023. Fig 6 is obtained by fitting TSV and PM2.5. The figure shows that when 10.2°C ≤ MPET<21°C, there is a clear fitting relationship between TSV and PM2.5. TSV gradually rises with the increase of PM2.5, which proves that there is interaction between TSV and PM2.5 in this range, consistent with the analysis results of SPSS software.

In general, through comprehensive analysis of SPSS software, the correlation between PM2.5 and MPET increased from P = 0.286 (10.2°C ≤ MPET ≤35°C) to P = 0.056 (10.2°C ≤ MPET<21°C). It can be concluded that when 10.2°C ≤ MPET<21°C, the correlation between MPET and PM2.5 is more significant, and the interaction between thermal environment and PM2.5 is stronger. And when 10.2°C ≤ MPET<21°C, P = 0.023 *, PM2.5 has a significant impact on TSV, as showed in Table 9.

### 3.2 Comprehensive impact on air quality evaluation

Linear regression analysis was used to analyze whether MPET and PM2.5 had significant effects on AQV and BCV. The research results are shown in Table 10. The research results show that MPET has no significant impact on both AQV and BCV, with P values of 0.135 and 0.251 respectively, while PM2.5 has a very significant impact on both AQV and BCV, with P values of 0.000 * * *, and the impact is significant at the level of 0.001. The data within the range of 10.2°C ≤ MPET<21°C were also analyzed by SPSS. The results are shown in Table 11. The research results show that PM2.5 has a very significant impact on AQV and BCV, with P values of 0.002 * * and 0.000 * *, respectively, which are significant at the level of 0.01 and 0.001. While MPET only has a significant impact on BCV, which is significant at 0.01 level, but has no impact on AQV, with P values of 0.01 * * and 0.056 respectively.

**Table 10:** The importance of indicators under air quality and breathing comfort.

		MPET	PM2.5
AQV	P	0.135	0.000***
	R2	0.114	0.672
	F	2.44	38.912
	equation	y = 0.0162x - 1.108	y = -0.0094x - 0.0191
BCV	P	0.251	0.000***
	R2	0.069	0.751
	F	1.401	57.297
	equation	y = 0.0089x -0.6069	y = -0.007x + 0.1465

**Table 11:** The importance of air quality and breathing comfort index when 10.2°C ≤ MPET<21°C.

		MPET	PM2.5
AQV	P	0.056	0.002**
	R^2^	0.253	0.529
	F	17.858	3.557
	equation	y = -0.0281x-0.4469	y = -0.0068x - 0.2833
BCV	P	0.01**	0.000***
	R^2^	0.414	0.689
	F	4.403	14.574
	equation	y = -0.0288x-0.0413	y = -0.0062x + 0.0574

**Fig 7:**
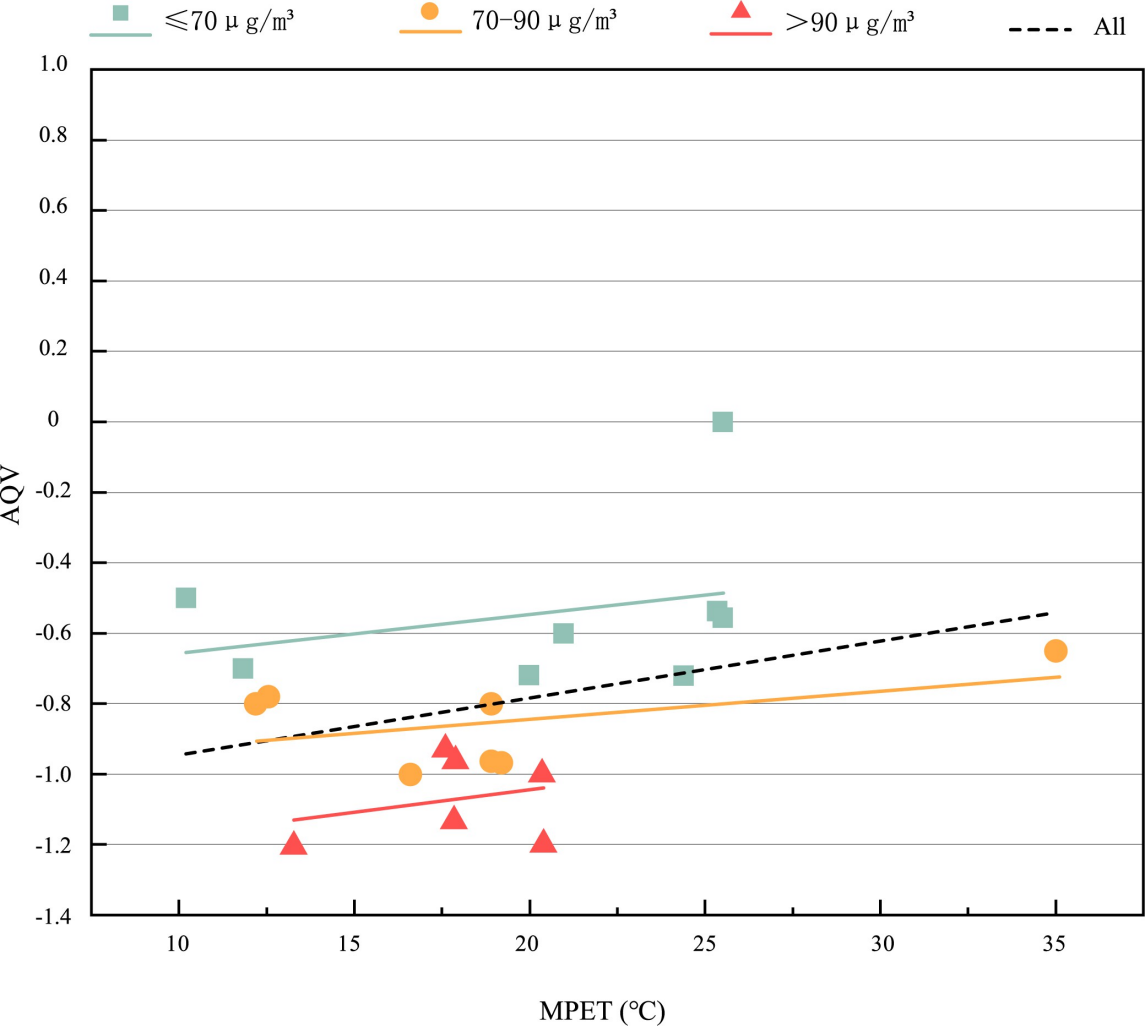
AQV under different thermal—PM2.5 environments.

**Fig 8:**
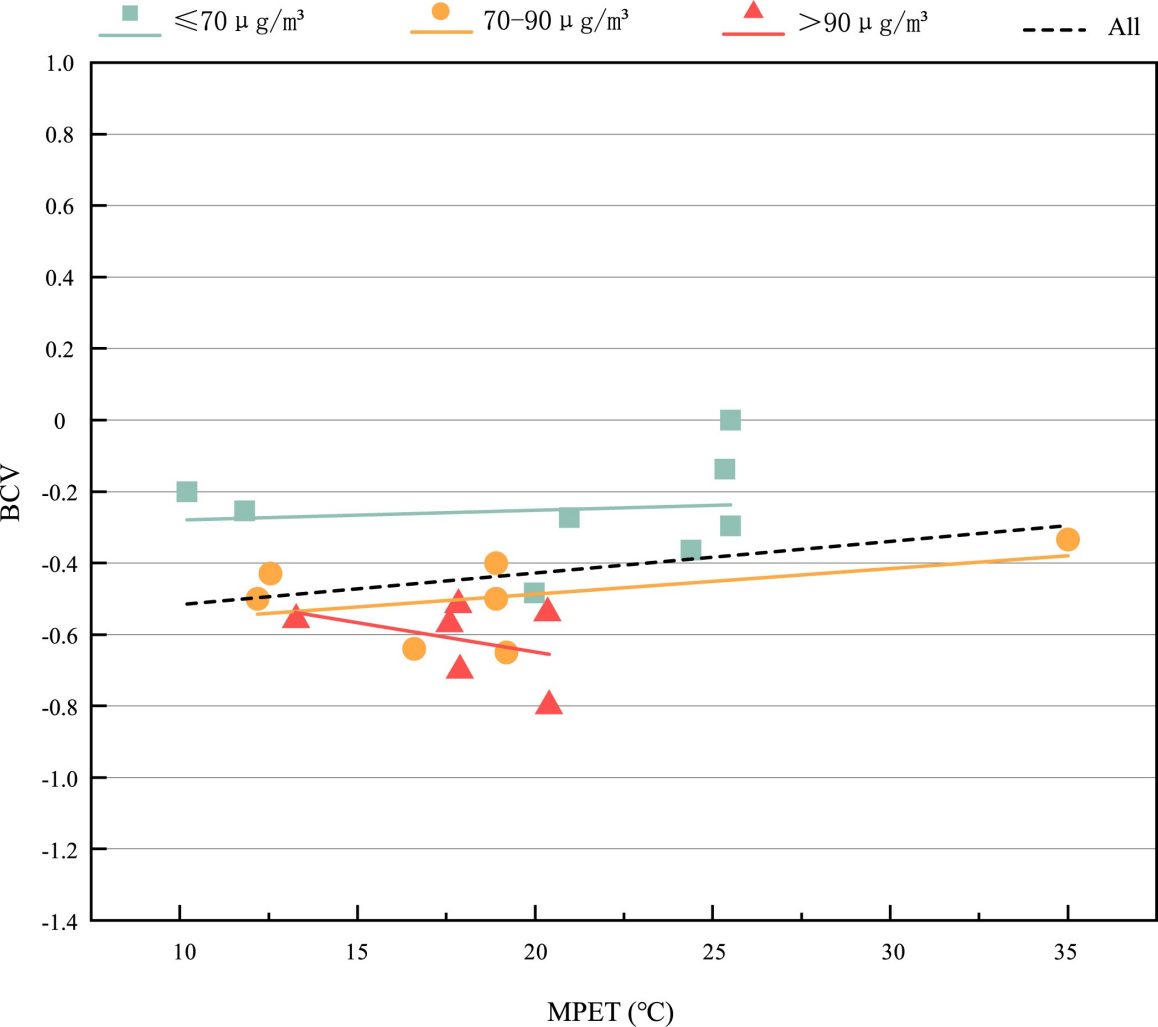
BCV under different thermal—PM2.5 environments.

Fig 7 shows the subjective AQV of the respondents in the thermal-PM2.5 environment. The range of MPET is 10.2°C~35°C, and the value of AQV is—1.2~0. The respondents’ subjective vote on air quality is almost all less than zero, with an average value of -0.89, indicating that the respondents are generally dissatisfied with the air quality in the current environment. Besides, we can find that there are obvious differences between different PM2.5 groups. When the MPET is the same, the worse the air quality is, the lower the AQV is. The average value of AQV under the environment of ≤70μg/*m*^3^, 70~90μg/*m*^3^ and >90μg/*m*^3^ is -0.54, -0.85 and -1.07 respectively, with a trend of decreasing. It shows that the subjective air quality perception of the respondents decreases with the increase of PM2.5. Fig 8 shows the subjective BCV of the respondents in the hot-PM2.5 environment. The range of MPET is 10.2°C~35°C, and the BCV value is -0.8~0. The respondents’ subjective vote on breathing comfort is almost all less than zero, with an average of -0.435, indicating that the respondents generally feel a little uncomfortable. Besides, we can find that there are obvious differences between different PM2.5 groups. When the MPET is the same, the higher the PM2.5 is, the lower the BCV is. The average value of BCV under the environment of ≤70μg/*m*^3^, 70~90μg/*m*^3^ and >90μg/*m*^3^ is -0.25, -0.49 and -0.61 respectively, with a trend of decreasing, which shows that the subjective breathing comfort of the respondents decreases with the increase of PM2.5. Moreover, we can observe that when 10.2°C ≤ MPET<21°C, PM2.5>90μg/*m*^3^ is different from that of other two groups. BCV decreases with the gradual increase of MPET, indicating that when 10.2°C ≤ MPET<21°C, PM2.5>90μg/*m*^3^, the increase of MPET will aggravate the uncomfortable feeling of breathing, and the thermal environment has a significant impact on the air evaluation index.

**Fig 9:**
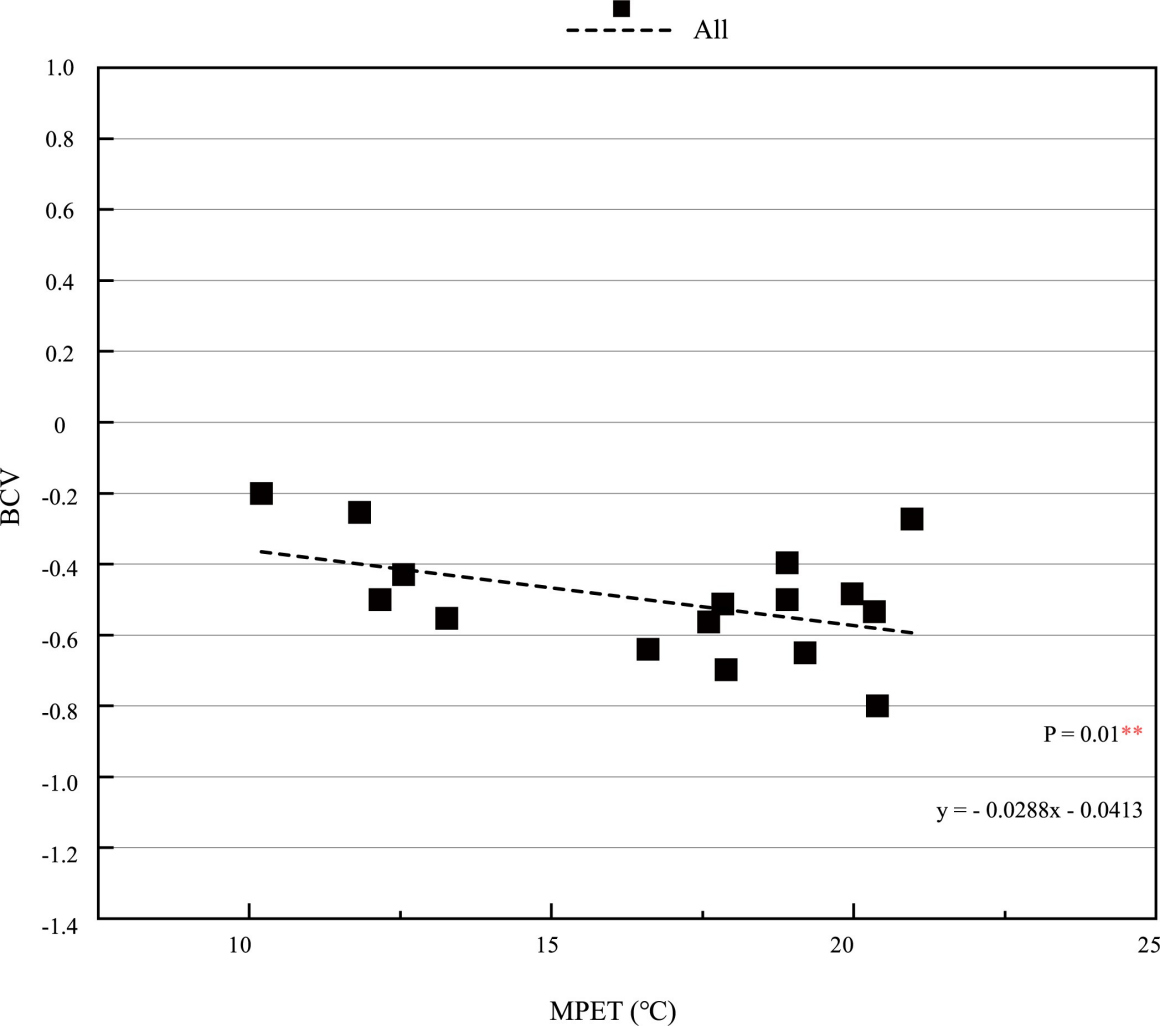
BCV under different thermal-PM2.5 environments when 10.2°C ≤ MPET<21°C.

The interaction between thermal environment and PM2.5 has been further studied. According to the uniqueness of PM2.5>90μg/*m*^3^ grouping, there are two kinds of conjectures about the fitting situation within the group: one is that the range of MPET is different from that of other groups, and the other is the concentration of PM2.5 is different from that of other groups. Therefore, based on the conditions of 10.2°C ≤ MPET<21°C and PM2.5>90μg/*m*^3^, the corresponding data were selected for correlation analysis with SPSS. The correlation P values of BCV and MPET are 0.01 * * and 0.451, respectively, the former is significant at 0.01 level while the latter is not, which shows that the special fitting condition in group is 10.2°C ≤ MPET<21°C instead of PM2.5>90μg/*m*^3^.The results are consistent with the critical value MPET = 21°C found in the previous comprehensive evaluation of thermal environment. The BCV and MPET in the range of 10.2°C ≤ MPET<21°C are fitted to obtain Fig 9. The figure shows that when 10.2°C ≤ MPET<21°C, there is a clear fitting relationship between BCV and MPET. The BCV gradually decreases with the increase of MPET, which proves that there is interaction between BCV and MPET in this range, which is consistent with the analysis results of SPSS software.

In general, through comprehensive analysis of SPSS software, the correlation between PM2.5 and MPET increased from P = 0.286 (10°C ≤ MPET≤35°C) to P = 0.056 (10.2°C ≤ MPET<21°C). It can be concluded that when 10.2°C ≤ MPET<21°C, the correlation between MPET and PM2.5 is more significant, the interaction between thermal environment and PM2.5 is stronger. And when 10.2°C ≤ MPET<21°C, P = 0.01 * *, MPET has a significant impact on BCV, as showed in Table 11.

### 3.3. Comprehensive impact on overall comfort evaluation

Linear regression analysis was used to analyze whether MPET and PM2.5 had significant effects on OCV. The research results are shown in Table 12. The research results show that MPET has a significant impact on OCV, with a P value of 0.041 *, which is significant at the level of 0.05, while PM2.5 has no significant impact on OCV, with a P value of 0.815. The data within the range of 10.2°C ≤ MPET<21°C were analyzed by SPSS. The results are shown in Table 13. The research results show that MPET has a very significant impact on OCV, with a P value of 0.001 * *, which is significant at the level of 0.001, while PM2.5 still has no significant impact on OCV, with a P value of 0.082.

**Table 12:** Importance of indicators under overall comfort.

		MPET	PM2.5
OCV	P	0.041*	0.815
	R^2^	0.202	0.003
	F	4.797	0.056
	equation	y = -0.0239x +0.0327	y = -0.0007x - 0.3705

**Table 13:** Importance of indicators under overall comfort when 10.2°C ≤ MPET<21°C.

		MPET	PM2.5
OCV	P	0.001***	0.082
	R^2^	0.579	0.215
	F	17.858	3.557
	equation	y = -0.0672x +0.7161	y = -0.0069x + 0.2391

**Fig 10:**
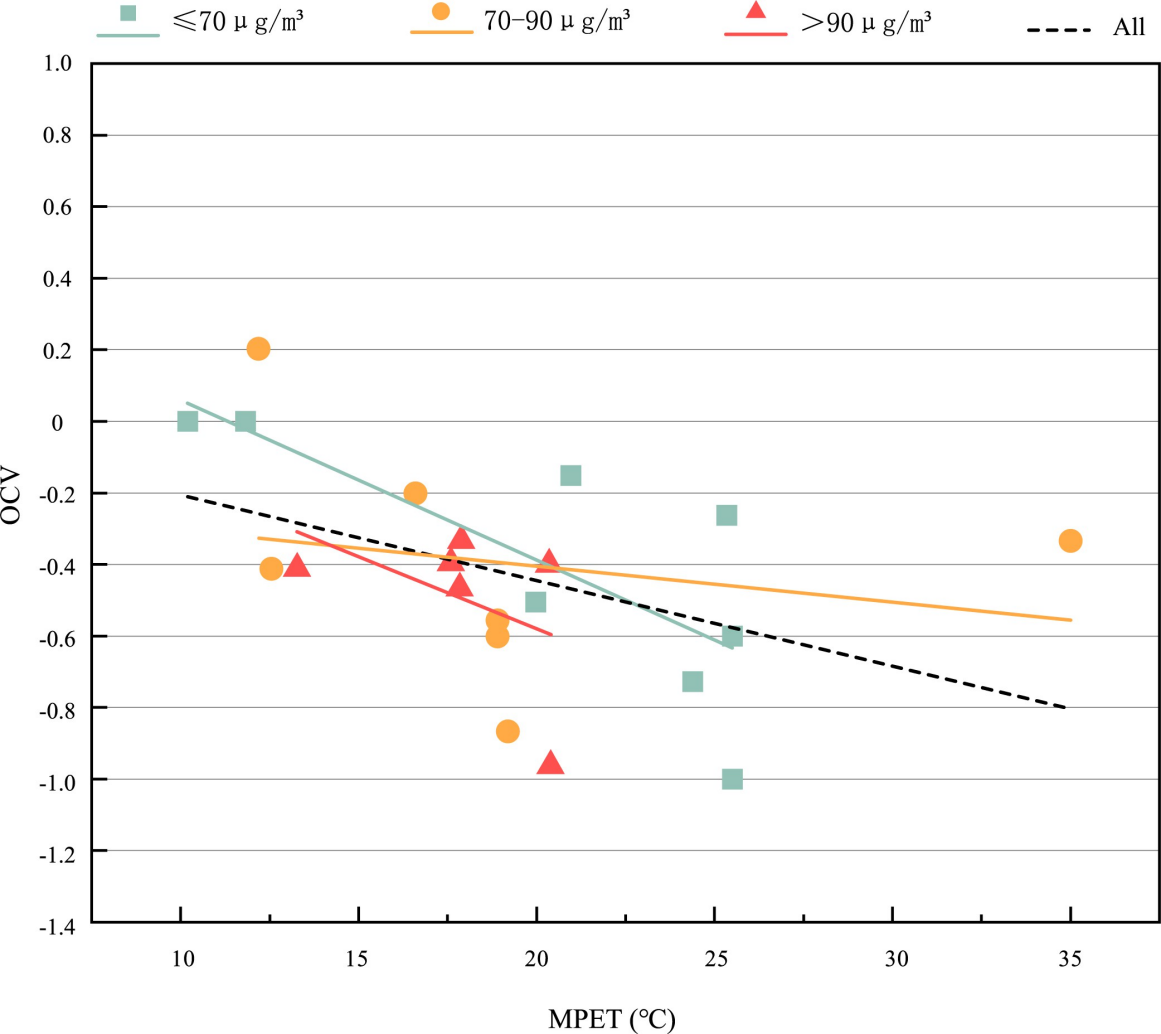
OCV voting values under different thermal-PM2.5 environments.

Fig 10 shows the subjective OCV of the respondents in the thermal-PM2.5 environment. The range of MPET is 10.2°C~35°C, and the OCV value is—1~0.2. The respondents’ subjective vote on the overall comfort is almost all less than zero, with an average of -0.43, indicating that the respondents generally feel a little uncomfortable about the overall comfort. In general, OCV gradually decreased with the increase of MPET. It is speculated that in April and May of the study, the weather was gradually hot, and the overall comfort of the respondents was significantly affected by MPET. Besides, we also found that in the range of about 10.2°C ≤ MPET<21°C, when people’s subjective thermal comfort is relatively moderate (the range of TCV is -0.30~0.13). The average value of OCV under the environment of ≤70μg/*m*^3^, 70~90μg/*m*^3^ and >90μg/*m*^3^ is -0.16, -0.4 and -0.5 respectively, with a trend of decreasing, which shows that the increase of PM2.5 will reduce OCV to a certain extent.

In order to further study the interaction between heat and PM2.5, the data in the range of 110.2°C ≤ MPET<21°C were analyzed by SPSS. The results showed that the impact of MPET on OCV increased from P = 0.041 * (10°C ≤ MPET≤35°C) to P = 0.001 * * (10.2°C ≤ MPET<21°C) compared with 10°C ≤ MPET≤35°C. The impact of PM2.5 on OCV also increased compared with 10°C ≤ MPET≤35°C, from P = 0.815 (10°C ≤ MPET≤35°C) to P = 0.082 (10.2°C ≤ MPET<21°C), but the impact of PM2.5 on OCV was not significant.

In general, MPET is a significant factor affecting OCV at any temperature, while PM2.5 has no significant impact on OCV. However, when 10.2°C ≤ MPET<21°C, the impact of PM2.5 on OCV is significantly increased.

## 4. Discussion

This paper focuses on the impact of the interaction of thermal environment and PM2.5 in urban outdoor space on human subjective comfort, and takes Xi’an Vocational University of Information as the research area. At present, many studies have been carried out on the thermal and air quality environment respectively, but few studies have been conducted on the interaction between heat and PM2.5. The subjective comfort of human body is affected by multiple factors and presents a complex situation. The subjective comfort of human body under the influence of multiple factors will be different from that under the influence of individual factors. In view of this, this study hopes to further explore the changes of human subjective comfort under the interaction of heat and PM2.5, and provide reference for the follow-up study of multi-environment interaction.

### 4.1 Thermal-PM2.5 interaction

Yin et al. found that in the afternoon, PM2.5 concentration is 0~20 μg/*m*^3^ and 20~40μg/*m*^3^, PM2.5 has a significant impact on TSV and TCV (p<0.001). The reduction of PM2.5 concentration can alleviate the thermal sensation of summer population, thus enhancing subjective thermal comfort [30]. The research above focuses on the impact of PM2.5 concentration in the morning, middle and evening of the day on subjective thermal evaluation. In this research, the average PM2.5 concentration is obtained by day, which can better reflect the daily average difference of PM2.5 concentration than by time period classification. Both our study and the study above have concluded that PM2.5 has a significant impact on TSV under certain conditions. The premise of the research above is that PM2.5 is between 0 ~ 20 μg/*m*^3^ and 20~40 μg/*m*^3^, the premise of our study is within the range of 10.2°C ≤ MPET<21°C. Our study believes that whether PM2.5 has a significant impact on TSV depends on whether it is in a relatively moderate thermal environment. The study above also pointed out that under constant physiological equivalent temperature (PET), air quality comfort decreases with the increase of PM2.5 concentration, and OCV will be relatively high when PM2.5 concentration is low [30]. It is similar to the conclusion of our study. The conclusion of our study shows that PM2.5 always has a significant impact on AQV and BCV (P = 0.000 * *), and both AQV and BCV have a negative correlation with PM2.5. Although the impact of PM2.5 on OCV is not significant, when 10.2°C ≤ MPET<21°C, the impact of PM2.5 on OCV increases, the average value of OCV under the environment of ≤70μg/*m*^3^,70~90μg/*m*^3^ and >90μg/*m*^3^ is -0.16, -0.4 and -0.5 respectively, with a trend of decreasing.

### 4.2 Interactions related to thermal environment

In urban outdoor space, subjective thermal evaluation of human body is affected by many factors. In this study, we found that when 10.2°C ≤ MPET<21°C, PM2.5 has a significant impact on TSV (Table 8, Fig 3, Fig 5), and it also has an influence on TCV. The average value of TCV under the environment of ≤70μg/*m*^3^,70~90μg/*m*^3^ and >90μg/*m*^3^ is -0.025, -0.127 and -0.160 respectively, with a trend of decreasing. Therefore, reducing the concentration of PM2.5 helps to improve subjective thermal comfort in the human body. We found similar conclusions in other studies on thermal environment interaction. Zhang et al. found that emotion can interact with the thermal environment. After the stress task, the subjects’ neutral PET, neutral PET range, preferred PET and thermal acceptable range changed from 19.4°C, 12.2~26.6°C, 25.2°C and 12.9~33.9°C to 16.3°C, 8.1~24.5°C, 24.3°C and 11.2~33.5°C, respectively, and after recovery, they were 20.0°C, 12.7~27.3°C, 25.3°C and 14.1~36.3°C [3]. This study shows that emotional changes will affect TSV. When designing and evaluating the thermal environment in the early stage, we can combine the function of the site and take into consideration the emotional factors of people in the site in the future using the space. Nagano et al. found that the thermal and acoustic environment will interact with each other when studying the comprehensive impact of cold and noise on human psychological stress. And the thermal and acoustic environment can affect overall comfort [31]. Jin et al. found that the interaction between thermal and acoustic environments occurs. The low temperature in winter and high temperature in summer will increase the acoustic discomfort. The acoustic environment will only affect the TSV in summer. When the UTCI is 21.6~40.4°C, the higher traffic noise will increase the thermal sensation. At the same time, the acoustic environment also has an impact on TCV. The increase of traffic noise leads to the reduction of TCV in three seasons [6]. The above two studies show that comfortable acoustic environment can increase the subjective evaluation value of urban outdoor space. Yan et al. took the urban pedestrian street in the extremely cold region of China as the research site, it was found that there was a significant positive correlation between the thermal comfort vote and the visual comfort vote. At the same time, it was found that people’s overall comfort was affected by the interaction of visual and thermal environment [32]. In addition, Lam et al. took Zhuhai, Guangzhou, China, as the research site, it was also found that there was a positive correlation between outdoor thermal sensation and sunlight sensation [33]. The above two studies confirmed the interaction between vision and thermal perception, indicating that a comfortable visual environment can help improve the subjective thermal evaluation value of urban outdoor space. Based on the above research on the interaction of multiple groups of thermal and other environments, we found that under the influence of the interaction of multiple environments, the improvement of other elements is conducive to better human subjective thermal evaluation. Therefore, our study believes that reducing the concentration of PM2.5 can improve the subjective thermal evaluation value of urban outdoor space, and outdoor space with good air quality can better the thermal comfort of teachers and students, thus promoting a series of associated efficiency, emotion, etc. We suggest that the outdoor space on campus can reduce the concentration of PM2.5 by increasing the green space rate and other ways to improve the subjective thermal evaluation value.

### 4.3 Interaction within the range of 10°C ≤ MPET<21°C

The correlation analysis results of SPSS within the range of 10.2°C ≤ MPET ≤ 35°C and 10.2°C ≤ MPET<21°C showed that when 10.2°C ≤ MPET < 21°C, TSV and PM2.5 were significant at the 0.05 level (P = 0.023 *), while MPET and BCV were significant at the 0.01 level (P = 0.01 * *).

**Fig 11:**
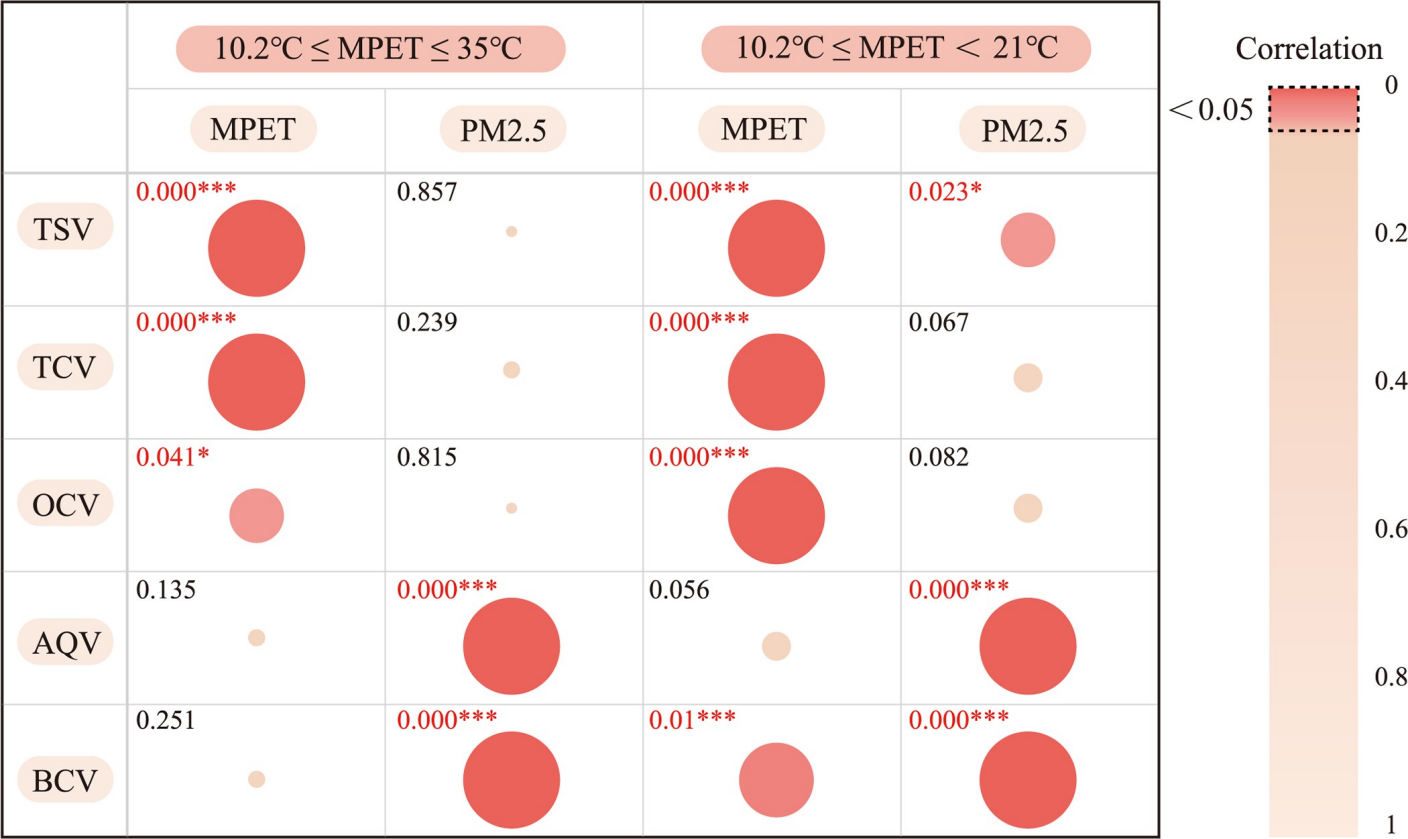
SPSS correlation analysis results within the range of 10.2°C ≤ MPET ≤ 35°C and 10.2°C ≤ MPET<21°C.

Fig 11 reports the SPSS correlation analysis results within the range of 10.2°C ≤ MPET ≤ 35°C and 10.2°C ≤ MPET<21°C. According to the research, the relatively moderate thermal environment (10.2°C ≤ MPET<21°C) is the key to determine whether some thermal and air quality indicators have significant correlation. The following conjectures are put forward: breathing When 10.2°C ≤ MPET<21°C, -0.5<-0.3 ≤ TCV ≤ 0.13<0.5, the TCV of the respondents is higher, and the impact of MPET on TSV and OCV is relatively small. So, respondents are more likely to distinguish the impact of PM2.5 on TSV; Respondents are more likely to feel the impact of MPET on BCV; It is also easier to distinguish the impact of PM2.5 on OCV. Therefore, when 10.2°C ≤ MPET<21°C, the correlation between thermal environment and PM2.5 is stronger.

### 4.4 Highlights

1. It was confirmed that thermal–PM2.5 environmental interactions and its impact on subjective comfort of the human body.
2. It was found that when 10.2°C ≤ MPET<21°C, thermal comfort is high, the thermal–PM2.5 environmental interactions shows significant effects on TSV and BCV. The study suggests that reducing PM2.5 concentration helps to improve thermal comfort, and improving thermal comfort helps to better breathing comfort.
3. The research finds that although both thermal environment and PM2.5 are important factors influencing OCV, thermal environment has a stronger impact on OCV.

### 4.5 Limitations

During the experiment, the range of Ta was from 13° C to 34° C. The study was conducted from April to May when the temperature was relatively mild. We concluded that the interaction between thermal environment and PM2.5 was more significant when the thermal comfort was at -0.5≤TCV≤0.5 (10°C ≤ MPET<21°C). Further research can be conducted in winter and summer when thermal comfort is lower, to improve the scientific and credible conclusions of the study. Meanwhile, our research was conducted on campus, with most participants being teachers and students. Further research can improve the universality of research conclusions by expanding the scope of the survey subjects.

## 5 Conclusion

Through questionnaire survey and meteorological parameter measurement, this study analyzes the impact of the interaction of thermal environment and PM2.5. Besides, this research attempts to determine how the interaction of thermal-PM2.5 environment affect the thermal comfort, breathing comfort and overall comfort. The data not presented in the text of this study are all presented in S1 Table. The main conclusions and corresponding recommendations of the study are as follows:

1. In terms of thermal environment, MPET has a significant impact on TSV and TCV, with P values of 0.000 * * *.PM2.5 has no significant impact on TSV and TCV when 10°C ≤ MPET ≤ 35°C, but has significant impact on TSV when 10.2°C ≤ MPET<21°C, P value is 0.023 *. This study finds that reducing PM2.5 concentration can improve the subjective thermal comfort value of urban outdoor spaces. So, we suggest that urban outdoor spaces can reduce PM2.5 concentration and improve subjective thermal comfort values at the same time by increasing green space ratio and installing sprinkler facilities.
2. In terms of air quality assessment, PM2.5 has a significant impact on AQV and BCV, with P values of 0.000 * * *. AQV and BCV have a negative correlation with PM2.5. MPET has a significant impact on BCV when 10.2°C ≤ MPET<21°C, P value is 0.01 * *. This study finds that improving the thermal environment helps to improve the subjective breathing comfort value of urban outdoor spaces. So, we suggest improving thermal comfort by installing shading facilities, water-cooled fans, and other methods, while also improving breathing comfort. Moreover, when dealing with patients with sudden symptoms such as shortness of breath outdoors, in addition to necessary scientific treatment, we can also help alleviate their symptoms by creating a more comfortable thermal environment.
3. In terms of overall comfort evaluation, MPET is a significant factor affecting OCV, while PM2.5 is not. However, when 10.2°C ≤ MPET<21°C, the impact of PM2.5 on OCV increases, from P = 0.815 (10°C ≤ MPET ≤ 35°C) to P = 0.082 21 (10.2°C ≤ MPET<21°C). In this study, although both thermal environment and PM2.5 are important factors affecting OCV, the former has a stronger influence. Improving the thermal environment and reducing PM2.5 both contribute to the improvement of overall comfort, which is the result of multiple factors working together.

## 6 Future work

In this paper, the interaction between thermal environment and PM2.5 has been studied. In the future, we will conduct in-depth research on the interaction between sound, light, heat, air quality and other multiple environments.

## Supporting information

S1 Table Data. (XLSX)
